# First person – Carina Lund

**DOI:** 10.1242/dmm.044362

**Published:** 2020-03-13

## Abstract

First Person is a series of interviews with the first authors of a selection of papers published in Disease Models & Mechanisms (DMM), helping early-career researchers promote themselves alongside their papers. Carina Lund is first author on ‘Characterization of the human GnRH neuron developmental transcriptome using a *GNRH1*-TdTomato reporter line in human pluripotent stem cells’, published in DMM. Carina is a PhD student in the lab of Taneli Raivio at the University of Helsinki, Finland, investigating neuronal differentiation from human pluripotent stem cells.


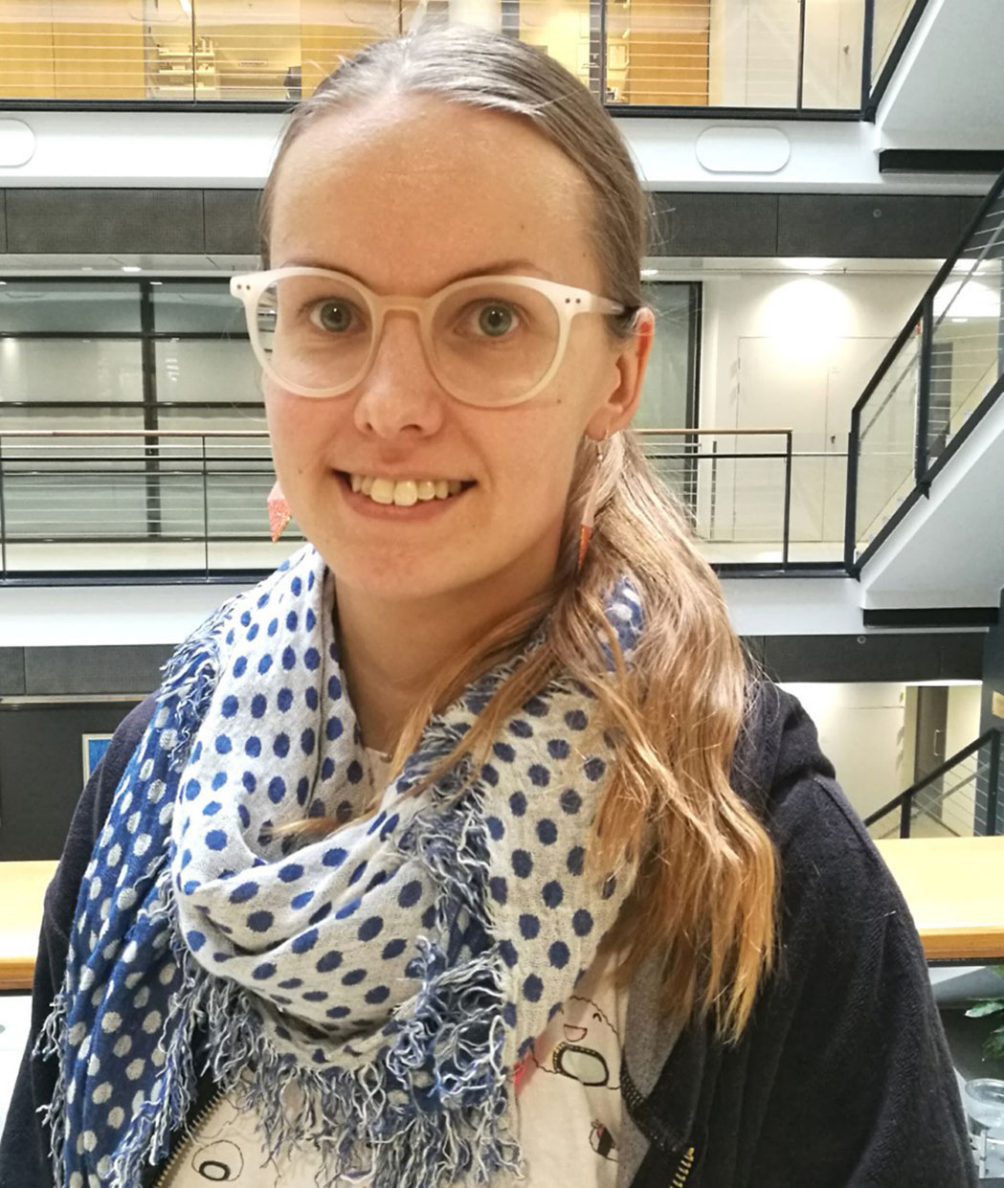


**Carina Lund**

**How would you explain the main findings of your paper to non-scientific family and friends?**

At puberty, the gonadotropin-releasing hormone (GnRH) neurons increase their pulsatile secretion of gonadotropin releasing hormone. These cells have a particularly interesting embryonic origin: they first emerge in the nasal area and form a group of cells migrating toward their final destination in the hypothalamus, a part of the brain with an important role in regulating many functions in the body. However, it is not well known how the GnRH neurons are born. GnRH deficiency is seen in a subset of rare genetic diseases that lead to delayed or absent puberty (congenital hypogonadotropic hypogonadism; CHH). The link between GnRH neurons and olfactory development is also seen in a developmental disorder called Kallmann syndrome, in which CHH is combined with loss of the sense of smell. Kallmann syndrome is phenotypically complex and genetically heterogeneous; different gene mutations may affect neuronal development, migration or GnRH hormone secretion. The need for new approaches to study these processes has prompted us to use human pluripotent stem cells as a tool for GnRH neuron formation in a culture dish. In the current article, we were interested in finding genes and gene networks involved in GnRH neuron differentiation. We generated a reporter cell line that enabled us to see nuclei of cells that express the *GNRH1* gene under a fluorescence microscope, and collected these cells from the neuronal cultures. We then characterized the gene expression at two developmental stages, and found more than 6000 differentially expressed genes, including several genes implicated in Kallmann syndrome.

**What are the potential implications of these results for your field of research?**

Our data bring up many new interesting target genes, pathways and networks, and offer a good basis for future research in the field of GnRH neuron biology, and for disease modelling of Kallmann syndrome and congenital hypogonadotropic hypogonadism.

**What are the main advantages and drawbacks of the model system you have used as it relates to the disease you are investigating?**

One of the major advantages of working with human pluripotent stem cells is access to human material for modelling of development and disease. Using human pluripotent stem cells, we have unlimited access to source materials, and GnRH neurons can be more easily picked out and studied with the new reporter cell line. Now, we can monitor the GnRH neurons in live cell cultures as well. Modelling of complex developmental diseases by recreating the tissue environment *in vitro* is a challenge. Nonetheless, it gives us access to early developmental stages that otherwise would be very rare, and understanding the development is of great importance in order to understand the diseases affecting reproductive health.

“One of the major advantages of working with human pluripotent stem cells is access to human material for modelling of development and disease.”

**What has surprised you the most while conducting your research?**

Since starting my PhD, the embryonic origin of GnRH neurons has been the most interesting topic. What distinguishes these progenitors from others in the nasal area, and how are these cell fate choices made? I hope that the discovery of ISL1 in human GnRH neurons takes us closer to figuring this out. It was promising to find several puberty-associated genes among our data, which means that our *in vitro* model has great potential in disease modelling endeavours in the future.

**Figure:**
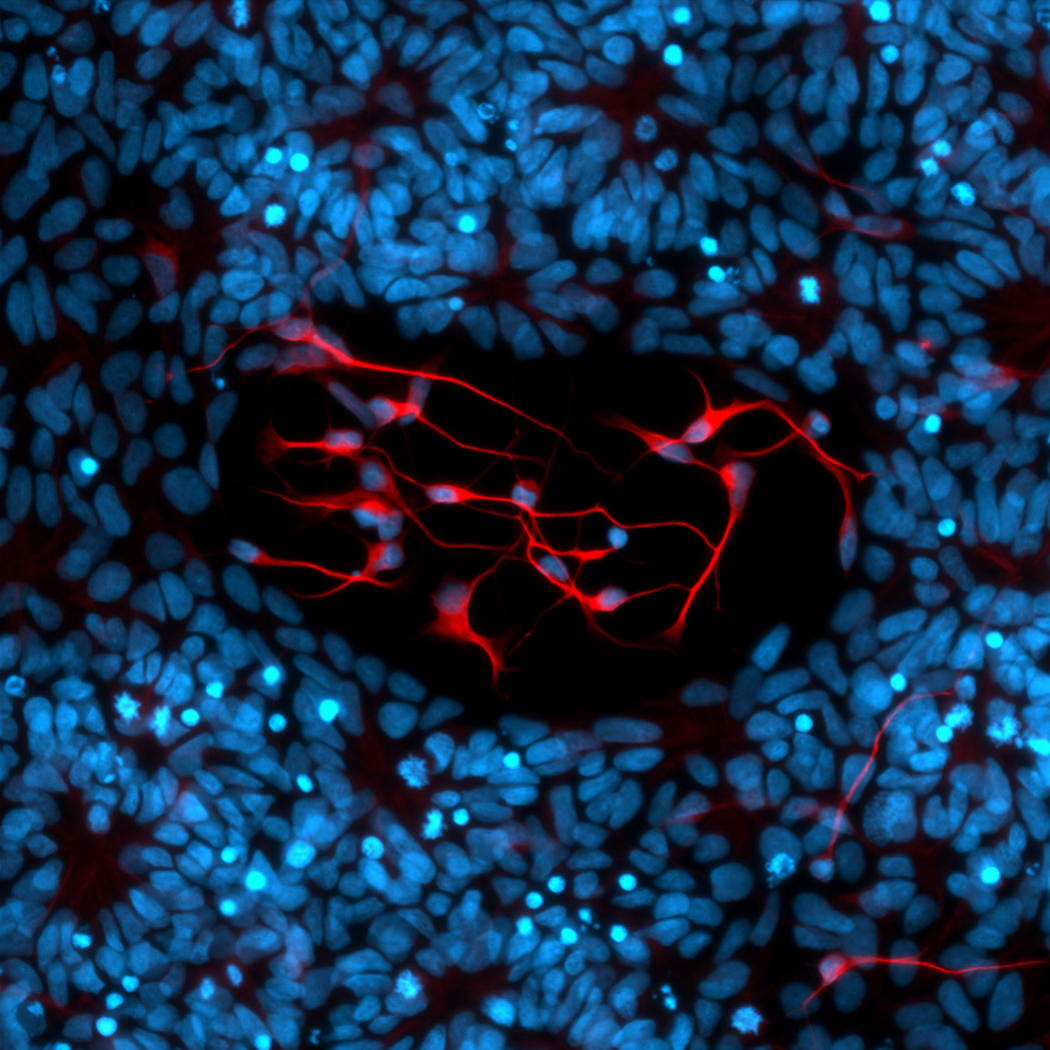
**Fluorescent image of differentiating neuronal cells at day 21, showing neurons in the centre and neuronal progenitors that form neuronal rosettes around the edges.** Once neurons have differentiated, they move out from the rosettes and grow long projections. Red, MAP2 antibody; blue, DAPI-stained nuclei.

**Describe what you think is the most significant challenge impacting your research at this time and how will this be addressed over the next 10 years?**

Tools for creating new models, such as genome-edited cell lines and omics-tools to study molecular aspects, have greatly improved in the last ∼10 years. The challenge is translation from gene to function, and from *in vitro* model to potential therapeutics. One approach would be to create better functional assays to investigate the molecular mechanisms, for example, in rare genetic diseases, developmental diseases, neurodegenerative diseases, etc.

**What changes do you think could improve the professional lives of early-career scientists?**

Mentoring and peer-support groups are not necessarily available for everyone at an early stage. Doctoral programmes could try to provide these kind of activities. It would be a good start towards making science and academia equal and inclusive. It is therefore important that research funding is not reduced. The possibility of employing more mid-level staff scientists at universities would likely improve the quality of research. For early-career scientists, it is important to have experienced, skilful people with lots of hands-on experience, and knowledge of all the tips and tricks.

“For early-career scientists, it is important to have experienced, skilful people with lots of hands-on experience, and knowledge of all the tips and tricks.”

**What's next for you?**

In 2020 I will defend my PhD thesis and start looking for new interesting projects. I am interested in developmental neuroscience, and hope to learn new techniques and research areas in this field, and perhaps get involved with other kinds of models, or focus on different diseases in the future.
